# The Burden of Pertussis Disease and Vaccination Coverage in Australian Adults Attending Primary Health Care

**DOI:** 10.3390/vaccines13101029

**Published:** 2025-10-02

**Authors:** Aye M. Moa, Juan C. Vargas-Zambrano, Hubert Maruszak, Valentina Costantino, C Raina MacIntyre

**Affiliations:** 1 Biosecurity Program, The Kirby Institute, Faculty of Medicine and Health, University of New South Wales, Sydney, NSW 2052, Australia; 2 Sanofi Vaccines Global Medical Affairs, 69007 Lyon, France; 3 Sanofi Australia & New Zealand Medical Affairs, Sydney, NSW 2000, Australia

**Keywords:** pertussis, vaccination, adults, primary health care, diagnostic testing, coughing illness

## Abstract

Background: The reported incidence of pertussis, a vaccine-preventable disease, has been increasing in recent years. This study aimed to estimate the burden of pertussis and the vaccination rate in Australian adults in primary care. Methods: Deidentified data for participants aged ≥18 years were extracted from the MedicalDirector (MD) primary care software from 2008 to 2019. We estimated the cumulative incidence of diagnosed pertussis in adults by age and risk groups and vaccine coverage in cases and a control group (not diagnosed with pertussis or a coughing illness). We also examined the incidence of unspecified coughing illness in the study population. Results: Of the 764,864 subjects included in the study, 1788 (0.2%) were diagnosed with pertussis between 2008 and 2019, corresponding to an average annual diagnosis rate of 76.9 per 100,000 population. About 31,110 (4.1%) of adults had an unspecified coughing illness. The highest rate was observed in 2011 and higher in females (63.3%), and the diagnosis rate was stable across all age groups. Underlying chronic conditions were more prevalent among pertussis cases than controls (58.7% vs. 18.8%), with asthma or chronic obstructive pulmonary disease (COPD) being the most common. Overall, 14% of cases received a pertussis vaccination during the study period. Diagnostic testing for pertussis was performed in 34.1% of pertussis cases. Estimated conservative costs per pertussis patient ranged from AUD 473 to AUD 909, with higher costs observed in individuals with complications. Conclusions: In the outpatient setting, there was a notable burden of pertussis among adults under 65 years of age, particularly those with underlying medical conditions, such as asthma and COPD, which appear to be significant risk factors. Due to the low rate of pertussis testing among all coughing illnesses, a proportion of non-specific coughing illness may be undiagnosed pertussis. The observed low vaccination rates highlight a need for increased awareness, improved diagnostic efforts, and prevention strategies in primary care.

## 1. Background

Pertussis is a vaccine-preventable disease that affects people of all ages, with the greatest severity observed in infants. However, older adults and people with chronic illnesses such as asthma or COPD have higher morbidity and mortality compared to healthy adults [1,2,3].

Generally, outbreaks of pertussis occur every 3–4 years, and prior to COVID-19, it was the second highest infectious disease notified to the National Notifiable Diseases Surveillance System (NNDSS) in Australia [4]. Australia has had among the highest reported incidence of pertussis globally in some years [5], with large outbreaks in 2011 and 2015, with an all-age notification rate of 173.3 and 94.8 per 100,000 population, respectively [4,6]. Between 2013 and 2018, the average all-age pertussis notification rate per year in Australia was 63.6 per 100,000 population [4], whilst in adults 65 years and over, the average rate ranged from 86 to 140 per 100,000 for the periods of 2006–2012 and 2013–2018 [4,6]. During 2020–2022, corresponding to COVID-19 mitigation measures, pertussis was much lower than pre-pandemic years; however, incidence has increased since 2023, with the largest epidemic ever reported in 2024, with an all-age notification rate of 215 per 100,000 population (over 57,000 cases), more than 70% of which occurred among older children and adults [7,8]. Continuing this trend, approximately 16,000 cases have already been reported in the first half of 2025 [8]. Pertussis immunity acquired from natural infection or vaccine-induced does not confer lifelong protection [9]. An increase in pertussis incidence has been reported in adults with underlying medical conditions in recent studies in Australia and other countries [3,10,11]. Adults may act as a reservoir of infection for infants and children; hence, timely diagnosis and treatment are vital for disease prevention.

Pertussis is notifiable in all states and territories in Australia. Both probable and confirmed pertussis cases are notifiable to the National Notifiable Diseases Surveillance System (NNDSS), and notifications are reported daily from each state or territory health department to the NNDSS [12]. According to Australian national notifiable diseases case definition, a confirmed case of pertussis is defined as the presence of laboratory definitive evidence or laboratory suggestive evidence and clinical evidence, while a probable case is defined as the presence of clinical and epidemiological evidence [13]. In contrast to children, the clinical presentation in adults can be atypical, often presenting with a non-specific or atypical cough, and general practitioners (GPs) may not test for pertussis [14,15,16]. Severe disease and deaths may occur in older adults, and outbreaks can occur in aged care facilities [17]. While deaths due to pertussis remained relatively low, pertussis mortality in adults 50 years and over were reported across the two surveillance periods, with 8 and 11 deaths during 2006–2011 and 2012–2018, respectively [4]. The identification of pertussis depends on health seeking by the patient, the GP’s awareness of the disease, and diagnostic testing [18]. Further, there are few GP consultations for pertussis vaccination and limited knowledge about the severity of the disease in adults [18]. Since 2000, polymerase chain reaction (PCR) is available for diagnostic testing in Australia, and in 2007, there were changes in reimbursement for PCR in the primary care setting. Testing practices may influence pertussis notifications over time; however, testing and awareness of pertussis is low in adults [19], with under-diagnosis and under-reporting by GPs [20]. Further, testing might not be conducted appropriately with either a PCR ordered too late or serology ordered too early, which may contribute to misdiagnosis.

In Australia since 2013, a booster dose of the diphtheria–tetanus–pertussis (dTpa) vaccine has been recommended but not funded for adults aged 50 years and ≥65 years if their last dose was more than 10 years ago or for any adult requesting the vaccine [21]. It is also recommended for at-risk populations such as health care workers, early childhood educators, and those providing care to children, including new parents. It is funded under the national program for all pregnant women (20–32 weeks) [21]. In Australia, the pertussis vaccine is only available in combination with tetanus and diphtheria for adolescents and adults [21]. Although opportunistic pertussis vaccination is recommended in the Australian Immunisation Handbook when a tetanus booster is required, the routine surveillance data showed that about one third of Australians receive dT instead of dTpa [22]. Pertussis vaccination in adults remains suboptimal nationally [23]. Data from the Australian Immunization Registry (AIR) in 2023 showed that 21% of adults aged 50 years or older were up-to-date for pertussis vaccination compared to tetanus and diphtheria vaccination, which was about 30% [22]. AIR is a national registry (passive surveillance system) for vaccination records for all people in Australia, with vaccines given under the NIP, school programs, and given privately such as trave vaccines [24], and adult vaccination is recorded in AIR from late 2016. Adult pertussis vaccination rates in Australia are lower than those in the United States, where Tdap vaccine coverage among adults aged ≥19 years was 26.6% overall in 2016, based on data from the National Health Interview Survey [25].

There is limited information on pertussis disease burden and the vaccination rate in primary care in Australian adults. This study aimed to determine the diagnosis of pertussis disease, associated complications, and the vaccination rate in Australian adults ≥18 years of age attending primary health care.

## 2. Methods

We conducted a retrospective cross-sectional study in patients ≥18 years of age attending the practices of consenting clinicians for a 12-year index period (2008–2019).

### 2.1. Data Source

Study data were extracted electronically using MedicalDirector (MD), a national clinical practice management software system used by just under half of all Australian GPs. As of December 2024, the database consisted of approximately 1300 users: primarily primary care practitioners (90–95%) and a smaller proportion of specialists. Participating clinicians had consented to participate and provided deidentified and aggregated patient data for research purposes. Geographically, the majority of data came from New South Wales and Victoria (~30–35%), followed by Queensland (<20%), and other states comprised < 10%. All analyses followed MD’s data governance model, which is underpinned by five tenets: integrity, safety, control, utility, and transparency. Data output was subject to minimum aggregation requirements, whereby no single cell of any output data was provided where the underlying data had 10 or fewer patients or patients from 3 or fewer medical practices due to privacy and confidentiality issues.

### 2.2. Study Population and Study Sample

The sample frame included all adults aged 18 years and over who had attended one of the primary care sites which use MD and where the clinician had consented. As patients are deidentified, if they attend more than one clinic, their records in each clinic appear as separate records. Deidentified, aggregated data was extracted electronically from participating primary care clinics over the study period (1 January 2008–31 December 2019), aimed to provide data broadly representative of the national primary care patient population.

To be eligible for the study, we identified a targeted sample of “active patients”, defined as those who have attended the same practice a minimum of 3 times in the previous 2 years, by using a definition from the Royal Australian College of General Practitioners [26]. The population at risk was considered as those active patients for each calendar year in the study. Among the active patients, three groups of patients were identified for data collection: (i) patients with a diagnosis of pertussis, (ii) patients with unspecified coughing illness without a pertussis diagnosis, and (iii) a control group. The selection criteria for each group are outlined below. Data were collected on clinical characteristics, sociodemographic factors, and vaccination history.

#### 2.2.1. Diagnosed Pertussis Cases

Pertussis cases were defined as patients who had a documented diagnosis of pertussis in their medical record in the study period, with or without laboratory confirmation of pertussis. If pertussis diagnosis was noted more than once for the same patient, then we only included the first diagnosis recorded in their patient file.

#### 2.2.2. Unspecified Coughing Illness

A secondary outcome was the incidence of coughing illness to determine the potential burden of coughing illness that may be related to undiagnosed pertussis in the study cohort attending primary care. Cases of coughing illness were defined as patients who did not have diagnosed pertussis and have had a coughing condition with no other diagnosis (as shown in the Supplementary Table S1) at any point during the study. Patients who have had coughing illness with a known, diagnosed condition were excluded (*n* = 5016 patients). These include gastro-oesophageal reflux disease (GERD), allergic rhinitis, chronic rhinitis, chronic sinusitis, interstitial lung disease, lung cancer, chronic cough (with asthma), chronic cough (with COPD), chronic cough (in patients who had been prescribed with angiotensin-converting enzyme (ACE) inhibitors), and coughing conditions due to any other respiratory infections, i.e., influenza or RSV.

#### 2.2.3. Controls

Controls were selected from active patients within the study period who had neither a diagnosis of pertussis nor any recorded coughing illness during that time.

### 2.3. Study Outcome and Data Analysis

Data analysis was restricted to active patients only according to the above-mentioned definition. The primary outcomes of interest were the cumulative incidence of pertussis or coughing illness in adults aged ≥18 years during the study period (2008–2019). Study data were analysed using descriptive epidemiology to compare three groups: patients diagnosed with pertussis, those with an unspecified coughing illness, and controls with neither condition. The overall study population and population subgroups were calculated using a denominator of all “active patients” (overall and within each subgroup). This allowed for the calculation of cumulative incidence for the overall population and each subgroup. Also, we stratified age by three groups, 18–44 years, 45–64 years, and ≥65 years. In addition, data were further analysed by at-risk conditions such as asthma, chronic obstructive pulmonary disease (COPD), cardiovascular disease (CVD), diabetes, and obesity. For analysis by states, there were some limitations of data regarding state and territory. There was missing data or limited information on post codes, and data were not available for Australian Capital Territory and Northern Territory due to values <10 counts in a cell.

Pertussis vaccination rate was estimated across all three groups among individuals who had received pertussis vaccine as adults at any point in the study period. The timing of vaccination was also estimated as being in the last 5 years (2015–2019) or earlier (2008–2014). In addition, patient records were reviewed to determine the timing of pertussis vaccination in relation to diagnosis, specifically, whether the vaccine was administered before or after the diagnosis. Data on other adult vaccines such as influenza, pneumococcal, and herpes zoster administered during the study period were also collected for comparison.

We determined the number of patients who had laboratory testing for pertussis using PCR or serology and the number of prescriptions issued for patients with pertussis and pertussis complications within 90 days of pertussis diagnosis (as shown in the Supplementary Table S2). We then estimated the number of general practice (GP) visits and associated cost of diagnosed pertussis. We determined costs associated with pertussis and pertussis complications in relation to GP visits, treatment, and additional medication costs among the at-risk population, and estimated the costs using information provided under the Australian government and the Pharmaceutical Benefits Scheme [27,28,29].

The costs of general practitioner consultations and pathology services for pertussis cases were estimated, incorporating both out-of-pocket expenses for patients and government contributions. Cost estimates were stratified by health care billing status (bulk-billed versus non-bulk-billed) and unit costs sourced from the Australian Government’s Medical Cost Finder [28,29]. Costs were calculated separately for patients with and without complications, and average annual costs were estimated during the study period.

Analyses were conducted using Microsoft Excel 2010. *p*-value was calculated using 2-proportion z-test where relevant, and odds ratio was calculated using a two-by-two contingency table and chi-square test. *p*-value of <0.05 was applied as the statistical significance. This study was approved by the Institutional Review Board (the University of New South Wales (UNSW HREC, Ref. HC220297).

## 3. Results

**Figure 1:**
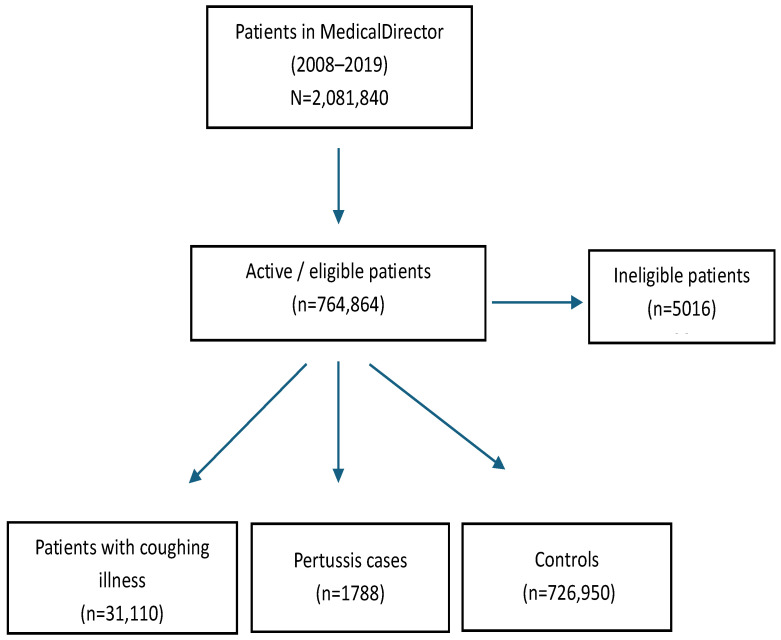
Consort diagram for the study.

A total of 764,864 active adult patients were selected for study from 2008 to 2019. Of these, 0.2% (1788/764,864) were diagnosed with pertussis, 4.1% (31,110/764,864) had an unspecified coughing illness, and 95% (726,950/764,864) had neither pertussis nor a coughing illness (controls) (Figure 1).

Of the pertussis cases, 33.7% (602/1788) were smokers, 58.7% (1049/1788) had comorbidities, and 4.4% (78/1788) of cases had pertussis complications. The overall demographic characteristics of participants are described in Table 1. All measured comorbidities (asthma or COPD, CVD, diabetes, and obesity) were significantly higher in pertussis cases compared to the controls, with asthma or COPD being the most prevalent comorbidity, occurring in a quarter of pertussis cases.

**Figure 2:**
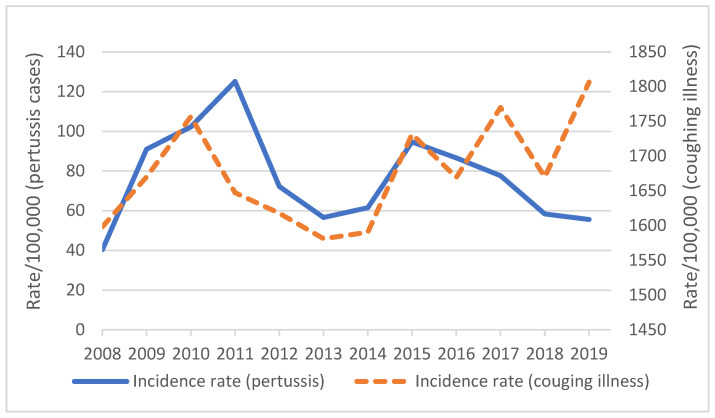
Diagnosis of pertussis and coughing illness rate per 100,000 population, 2008–2019.

The diagnosis rate of pertussis and coughing illness varied by year but followed a similar trend until the last 3 years, when the unspecified coughing illness rose (Figure 2). The all-age average annual diagnosis rate of pertussis was 76.9 per 100,000 population in adults >18 years. The highest rate was recorded in 2011 at 125.1 per 100,000, followed by 102.2 and 94.7 in 2010 and 2015, respectively.

**Figure 3:**
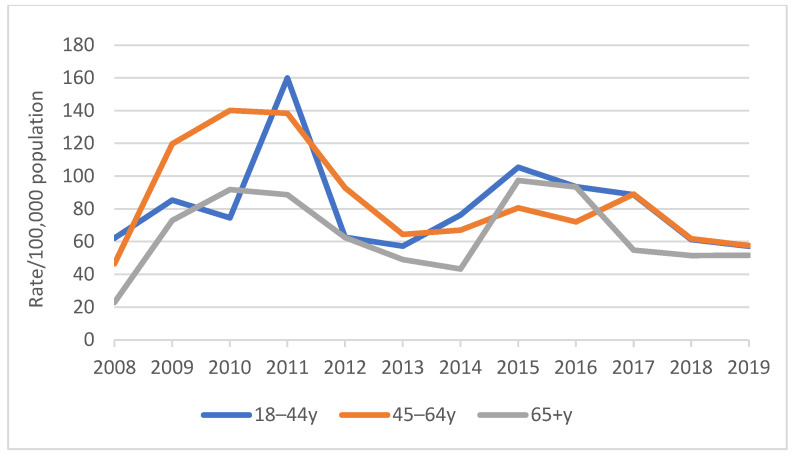
Diagnosis rate of pertussis by age group, 2008–2019.

The rate of diagnosed pertussis varied by age group, as shown in Figure 3; the highest rate was recorded in the 18–44-year age group, followed by the 45–64-year age group in both 2011 and 2015 in the study.

**Figure 4:**
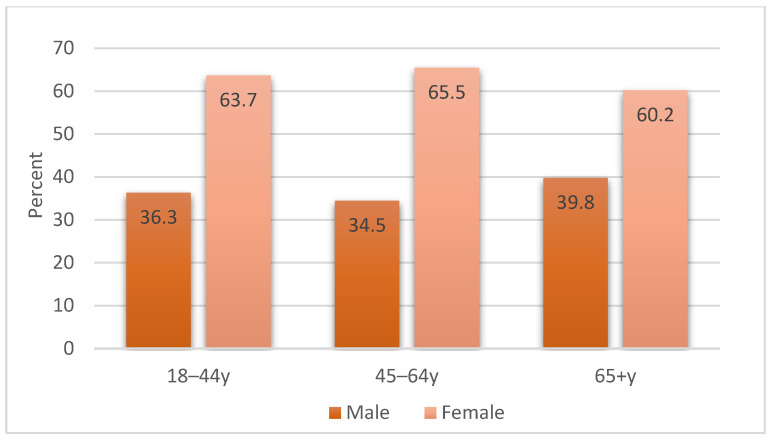
Distribution of pertussis cases by age group and sex, 2008–2019.

Among pertussis cases, females represented a higher proportion of cases with 63.3% (1131/1788) compared to males (36.7%, 657/1788) (Table 1) and an odds ratio (OR) of 1.31 (95%CI 1.19–1.44, *p* < 0.001). Distribution by age group and sex among cases is presented in Figure 4, where consistent findings were observed across the different age groups. Variation in pertussis diagnosis rates was observed across states and territories during 2008–2019. Western Australia reported the highest rate (936.7 per 100,000 population), followed by New South Wales and Queensland at a rate of 270.3 and 243 per 100,000, respectively.

### 3.1. Chronic Conditions or Other Comorbidities

About 59% of pertussis cases had comorbid conditions such as asthma/COPD or CVD or diabetes or obesity, with asthma/COPD being the most frequent (24.9%), followed by CVD, diabetes, and obesity (13.8%, 11.2% and 8.7%, respectively). Only 18.8% of controls had comorbidities. The presence of comorbidities among pertussis cases and controls by age group are presented in Table 2.

Cases were six times more likely to have had comorbidities compared to the controls, with an OR of 6.16 (95%CI: 5.61–6.77), *p* < 0.001. The odds ratio varied with various comorbid conditions among cases and controls, as presented in Supplementary Table S3.

### 3.2. Pertussis Complications

Of all the cases, 4.4% (78/1788) had pertussis complications, the most common being pneumonia, ranging from 15 to 45% of all complications across age groups. The presence of complications across various age groups is shown in Table 3.

The age group 45–64 years had a significantly higher rate of complications OR = 1.80 (95%CI: 1.01–3.19) compared to the 18–44 years. Similarly, the age group ≥65 years had a higher rate of pertussis complications compared to younger cases (18–44 years) with an OR of 1.50 (95%CI: 0.81–2.75), but this was not statistically significant (Table 3).

### 3.3. Vaccination Rate

Of all active patients, 31.3% (239,385/764,864) received any adult vaccination with either influenza, pertussis, pneumococcal, or herpes zoster vaccine during the study period.

**Figure 5:**
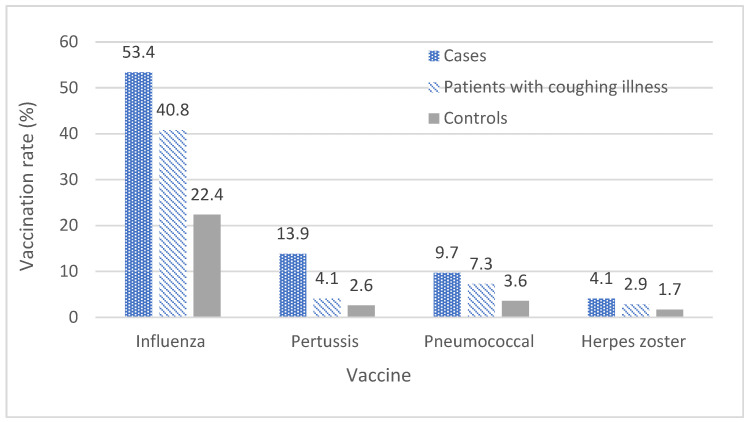
Vaccination rate by group and type of vaccine, 2008–2019, all ages.

Of pertussis cases, 13.9% (247/1782) were vaccinated against pertussis. Over 90% of cases in the study received their pertussis vaccine within the five-year period (2015–2019). Among the 247 vaccinated cases, 59% (145/247) received the vaccine prior to their pertussis diagnosis. While data were not reported due to data values of <10 per cell, less than 7% of cases received a pertussis vaccination after the diagnosis. Notably, pertussis vaccination rates were low in patients with a coughing illness and among controls, at 4.1% (1278/31,254) and 2.6% (18,881/730,094), respectively. Vaccination rates among cases, those with a coughing illness and the controls, by the type of vaccine are shown in Figure 5.

**Figure 6:**
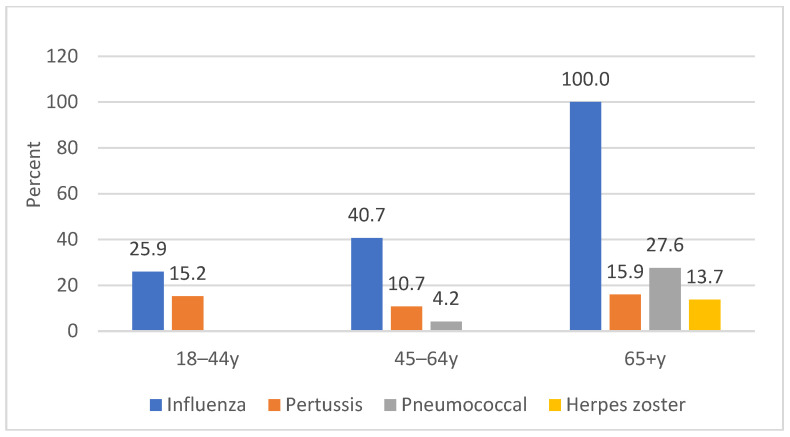
Vaccination rate in cases by age group and type of vaccine, 2008–2019.

Figure 6 shows the rate of vaccination in cases with various vaccines by age group. There was variation in vaccination rates across age groups, and the vaccine uptake was high in older adults ≥65 years for all vaccines.

### 3.4. Diagnostic Testing

**Figure 7:**
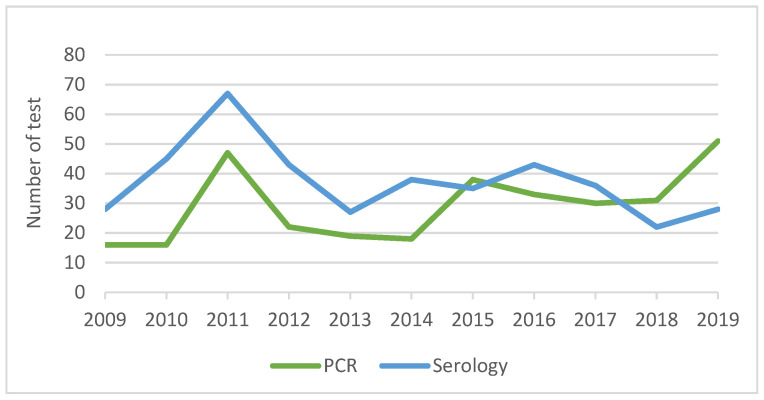
Use of diagnostic method in pertussis cases, 2009–2019. Year 2008 was omitted due to values of <10 per cell.

General practitioners requested a total of 609 laboratory tests for pertussis over the study period. There was year-to-year variation in the number of diagnostic tests performed during the study period (Supplementary Figure S1), and the testing trends closely mirrored the number of cases diagnosed over the years. The overall rate of laboratory testing for cases was 34.1%. Among pertussis cases, PCR and serology testing accounted for 57.5% and 42.5% of all tests, respectively. The rate of PCR and serology testing for pertussis among the coughing illness group was 65% (723/1113) and 35% (390/1113) of all tests, respectively. The use of diagnostic methods varied over time; a gradual increase in PCR testing was also observed (Figure 7).

### 3.5. Medications

Table 4 shows the prescription rate per 1000 patients who had comorbidities. Cases were more likely to have an increased use of medications compared to controls who had a similar comorbid condition, and that increases with age except for obesity medication (Supplementary Table S4).

Pertussis cases without complications had an average of nine GP visits related to pertussis, compared to twelve for those with complications. Table 5 presents the associated primary care costs for pertussis cases, including both patients’ out-of-pocket expenses and government-funded costs. Among patients without complications, the average estimated cost per patient ranged from AUD 473 to AUD 909. For those who developed complications, costs were higher, ranging from AUD 596 to AUD 1164 per case (Supplementary Table S5). The total estimated average annual cost of pertussis cases analysed in the study was approximately AUD 91,000, with higher overall costs observed among patients who experienced complications.

## 4. Discussion

We observed a substantial burden of adult pertussis in the Australian primary care setting over more than a decade, with incidence rates and trends closely aligning with national notification data. The all-age average annual diagnosis rate was 76.9 per 100,000 population, with notable epidemic peaks occurring in 2011 and 2015 [4]. According to the National Notifiable Diseases Surveillance System, the average all-age notification rate of pertussis in Australia was 89.4 per 100,000 population between 2008 and 2019 [8]. Similarly, an observational study conducted in Australian primary care from 2015 to 2019 reported pertussis incidence rates ranging from 57.6 to 91.4 per 100,000 among adults aged 50 years and older [3]. A descriptive study from the national surveillance program in Australia found that the average rate of pertussis in adults aged 65 years and over was 86 per 100,000 population from 2006 to 2012 [6]. Another large population-based cohort study from the state of New South Wales reported a pertussis incidence rate of 94 per 100,000 person-years in adults aged ≥ 45 years [30]. Like several other studies, we found that females had a higher incidence of pertussis [3,4,30]. The higher rate in women may reflect greater contact with children due to parental and grandparental care [31,32]. Like Australia, studies from other countries showed an increasing burden of pertussis in adults in recent years [1,33,34]. There was some variation in pertussis diagnosis rates across states and territories, with Western Australia reporting the highest rate during the study period. This pattern may reflect an increasing burden of disease or, alternatively, increased testing practices within the state and more broadly across Australia since 2007 [35]. Our findings in WA contradict previously reported data on diagnosis rates for a similar period [36]; however, the underlying reasons for the observed increase in cases remain unclear.

Asthma and COPD are one of the most common chronic conditions in Australia, and people with asthma/COPD are at an increased risk of pertussis infection and its complications [1,2,37,38]. One Australian study reported a pertussis incidence rate of 160 per 100,000 person-years among asthmatic adults aged ≥50 years, compared with 90 per 100,000 person-years in those without asthma [30]. A history of tobacco smoking and asthma was also associated with an increased risk of pertussis-related hospitalisation in adults aged >65 years [39]. Other studies have similarly identified asthma, COPD, immunosuppression, obesity, and smoking as important risk factors for pertussis [40].

Severe pertussis in adults can result in complications such as pneumonia, sinusitis, seizures, urinary incontinence, encephalitis, secondary respiratory infection, rib fracture, subdural haemorrhage, and even death [17,34,41]. We showed that less than five percent of pertussis cases in primary care developed complications, most commonly pneumonia. However, we did not capture data on hospitalisation or emergency department visits, which may be the first site of presentation for people with severe complications. This may explain why people aged > 65 years had a lower rate of complications compared to 45–64-year-olds in our study. This may be due to fewer presentations to GPs among older cases, who may have presented to emergency departments instead [39].

Over 85% of patients with pertussis were unvaccinated despite seeing a GP at least three times over two years, highlighting a critical missed opportunity for prevention. We observed low pertussis vaccination rates, even among older patients and those with comorbidities. The observed low vaccination rate may be influenced by several factors, including low awareness among GPs regarding adults pertussis and its severity, resulting in lower patient education regarding vaccination, as well as patient hesitancy towards vaccination in general [17,18,42]. In addition, the cost of vaccination may be a barrier as the pertussis vaccine is not funded under the national program for adults or at-risk populations, except for pregnant women [22]. Also, a lower uptake of dTpa compared to diphtheria–tetanus toxoid-containing vaccine (dT) in adults needing a tetanus booster is a factor, despite the Australia Immunisation Handbook recommending dTpa [21,22]. According to the Australian Immunisation Register, only about 21% of Australian adults aged 50 years and older were vaccinated against pertussis in 2023 [22]. Tetanus boosting is an important opportunity for pertussis vaccination. A recent study found that the tetanus vaccination rate was 30%, compared to just 20% for pertussis, which likely reflects the use of dT instead of dTpa for tetanus boosters [22]. In a 2009 vaccination survey, pertussis vaccine coverage in Australian adults was only 11.3% [43]. In this primary care setting, we found that only 11% of cases aged 40–64 years and 16% of those 65 years and over had received a pertussis vaccine. The majority of vaccinated cases had received the vaccine within the past 5 years (2015–2019); notably, 59% of cases were vaccinated prior to their pertussis diagnosis, while approximately 35% received the vaccine or were recommended vaccination only after their diagnosis. Low adult pertussis vaccine uptakes have been reported in other Australian studies [3,18]. The slightly higher vaccination rates observed among pertussis cases compared to controls may reflect a healthy user bias, where patients with chronic conditions or those who are at a higher risk of pertussis are more likely to seek health care services and receive vaccinations. However, further investigation is warranted to better understand this association. As well, it cannot be discarded that those that were recorded as unvaccinated might have received the vaccine somewhere else, and it is not yet recorded in the system.

Vaccine efficacy for the acellular pertussis vaccine in adults 15–65 years in a randomised, controlled trial in the US found a protection rate of 92% [44], and a meta-analysis of the pertussis vaccine in adolescents and adults mentioned a short-term effectiveness of the acellular pertussis vaccine, with vaccine response rates of >85% among the studies [45]. However, a low to moderate vaccine effectiveness (VE) of pertussis was reported in adults in studies [46,47]. Vaccine effectiveness can be dependent on age and the study setting and time since vaccination. A moderate vaccine effectiveness, an adjusted VE of 52%, was also noted in an Australian study for PCR-confirmed cases [46]. Authors of that study also reported a variation in VE by the timing of vaccination, and a non-significant VE of 42% (−23 to 73%) was found in cases if they were vaccinated between 2 to 5 years. More research is needed to evaluate the effectiveness of the pertussis vaccine in adults. Recommendations for adult pertussis vaccination vary across countries [40]. Nonetheless, in settings with a high disease burden, even a vaccine with moderate effectiveness can have a substantial impact on population health [48].

In addition to health impacts, pertussis leads to increased use of health services and creates an economic burden. A UK study found that, among adults aged ≥50 years, those with asthma had a higher health care utilisation, and their direct medical care costs per individual was increased by GBP 370 (95% CI 128–650; 26.4% higher) compared to people without asthma in the study [1]. Our costing did not include hospital costs, which are far higher than community costs per episode. Nonetheless, during an epidemic year, community costs of pertussis may be substantial. In 2024, Australia recorded the largest epidemic of pertussis with over fifty-seven thousand notified cases in the year [8]. If all these cases had seen a GP, based on our estimated costs per episode, the total cost incurred in primary care may have been at least around AUD 27 million dollars, excluding any hospitalisation costs.

The laboratory methods used to diagnose pertussis has varied over time. Consistent with our findings, the use of PCR has ranged from 50% to 90% of all tests compared to serology, which varied from 50% to 10% between 2014 and 2018 in Australia [4]. The availability and use of PCR testing for pertussis have been increased significantly since 2005 following the introduction of public funding for laboratories conducting PCR tests under the Medicare Benefit Schedule [49]. However, diagnostic testing for pertussis also depends on the GP’s recognition or awareness of pertussis as a differential diagnosis. Due to under-ascertainment and under-reporting, the true burden of pertussis in adults may be higher. Pertussis can present with a chronic or prolonged cough and is frequently under-ascertained or misdiagnosed in adult patients [50]. We found that 4.1% of patients had an unspecified coughing illness during the study period, and the majority had not been tested for pertussis. Improved awareness and testing for pertussis is needed. Another area for research is the burden of pertussis in aged care settings, where pertussis epidemics do occur; however, testing in these settings is mostly limited to viral PCR, and the awareness of pertussis may be even lower than in the community [51].

There were several limitations in the study. First, due to privacy, data were not provided in cells with values less than 10, resulting in missing data. We could not obtain specific data on complications, as the occurrence of individual complications other than pneumonia was less than 10 counts. This limitation may have led to an underestimation of disease severity within the study population. Second, data on pathology test results for the laboratory confirmation of cases could not be extracted by MD software, so we had to use tests ordered as a proxy. The third limitation was that, when an eligible patient attended two practices, the patient was counted as two distinct patients; thus, this could lead to double counting of active patients in the study population. Fourth, depending on the data we received in the study, multivariable analyses could not be performed using advanced analyses to control confounding variables or inherent sociodemographic differences between cases and controls were not possible. The odds ratio presented should be interpreted with caution. Furthermore, measures of vaccine protection or effectiveness should not be estimated from this study, as data were provided in aggregated form. Fifth, due to data limitations, our cost estimations were restricted to primary care services related to pertussis; as a result, these likely underestimate the overall economic burden by excluding expenses associated with emergency visits and hospitalisations. In addition, hospitalisations, which represent the most severe end of the spectrum of pertussis, were not captured in the MD dataset. Sixth, the data were not comprehensive of all outpatient visits, and some patients may have received vaccinations outside the study setting. Finally, although our study commenced in 2022, data from 2020 and 2021 were not included, as the number of cases reported nationally was very low, likely reflecting the impact of non-pharmaceutical interventions such as lockdowns and masks implemented during the COVID-19 pandemic [52]. This may limit the comparability of temporal trends in pertussis during recent years.

## 5. Conclusions

We found a substantial burden of pertussis in adults in primary care, with a higher incidence in people with comorbidities, all of which cause a health and economic burden to Australia, especially during high epidemic years. Asthma and COPD are the most important comorbid conditions in people with pertussis. Pertussis vaccine uptake in the primary care setting was very low, reflecting a missed opportunity for prevention. Diagnostic testing for pertussis was also low, and a proportion of unspecified coughing illness may be caused by pertussis, especially in epidemic years. GPs play an important role in vaccination, awareness, and testing for pertussis.

## Figures and Tables

**Table 1:** Characteristics of the study population, 2008–2019.

	Active/Eligible Patients in the Study * (N)	Diagnosed Pertussis **, *n*	Patients with Coughing Illness ***, *n*	Controls ^#^, *n*
Total number of patients	764,864	1788 (0.2%)	31,110 (4.1%)	726,950 (95.0%)
**Age group**				
18–44 y		614 (34.3%)	12,388 (39.8%)	298,497 (41.1%)
		***p*** **< 0.001**	***p*** **< 0.001**	Ref
45–64 y		626 (35.0%)	9629 (31.0%)	223,944 (30.8%)
		***p*** **< 0.001**	***p*** **= 0.603**	Ref
65+ y		548 (30.6%)	9093 (29.2%)	204,509 (28.1%)
		***p*** **= 0.017**	***p*** **< 0.001**	Ref
**Sex**				
Females		1131 (63.3%)	18,301 (58.8%)	412,645 (56.8%)
		***p*** **< 0.001**	***p*** **< 0.001**	Ref
**Smoking status**				
Subjects who were smokers		602 (33.7%)	n/a	229,067 (31.5%)
		*p* = 0.05		Ref
**Remoteness index ^##^**				
Live in major cities in Australia		1224 (68.5%)	n/a	n/a
**Comorbidity**				
Comorbidities (all)		1049 (58.7%)	n/a	136,603 (18.8%)
		***p*** **< 0.001**		Ref
Asthma or COPD		446 (24.9%)	n/a	31,856 (4.4%)
		***p*** **< 0.001**		Ref
CVD		251 (13.8%)	n/a	43,859 (6.0%)
		***p*** **< 0.001**		Ref
Diabetes		201 (11.2%)	n/a	39,718 (5.5%)
		***p*** **< 0.001**		Ref
Obesity		156 (8.7%)	n/a	21,170 (2.9%)
		***p*** **< 0.001**		Ref
**Laboratory testing for pertussis**				
Tests requested by GP (PCR or serology)		609 (34.1%)	1113 (3.6%)	n/a
		***p*** **< 0.001**	Ref	
**Pertussis complications**				
Complications (any) ^###^		78 (4.4%)	n/a	n/a
**Recorded rate of vaccination, adults all ages, 2008–2019**				
Vaccinated with pertussis vaccine		247 (14%)	1278 (4%)	18,881 (3%)
		***p*** **< 0.001**	***p*** **< 0.001**	Ref

* An active patient is defined as one who has attended the same practice a minimum of 3 times in the previous 2 years. ** Diagnosed pertussis, which included both clinical and lab-confirmed cases. *** Patients who do not have a diagnosis of pertussis during the year, but who have had one of the coughing conditions (Supplementary Table S1) at any point during the year. ^#^ Patients who had neither pertussis nor coughing illness during the study. ^##^ Remoteness index was applied from post codes to 2016 census data. ^###^ Patients who had pertussis during the year, and who have had one of the complications (Supplementary Table S2) within the 3 months of diagnosis. n/a, data was not applicable. COPD, chronic obstructive pulmonary disease; CVD, cardiovascular disease; GP, general practitioner; and PCR, polymerase chain reaction. Statistically significant *p*-values are in bold font. *p*-values were calculated using 2-proportion z-test. Data on ineligible patients (*n* = 5016) were excluded in the study.

**Table 2:** Pertussis cases and controls * who had comorbidities, by age group, 2008–2019.

Age Group	Patients, *n*	Asthma or COPD, *n* (%)	*p*-Value	CVD, *n* (%)	*p*-Value	Diabetes, *n* (%)	*p*-Value	Obesity, *n* (%)	*p*-Value
18–44 y									
Cases	614	120 (19.5)	**<0.001**	**	n/a	17 (2.8)	**0.014**	42 (6.8)	**<0.001**
Controls	298,497	7466 (2.5)		2083 (0.7)		4595 (1.5)		8440 (2.8)	
45–64 y									
Cases	626	141 (22.5)	**<0.001**	51 (8.1)	**<0.001**	60 (9.6)	**<0.001**	67 (10.7)	**<0.001**
Controls	223,944	8587 (3.8)		8603 (3.8)		12,788 (5.7)		8702 (3.9)	
65+ y									
Cases	548	185 (33.8)	**<0.001**	195 (35.6)	**<0.001**	124 (22.6)	**<0.001**	47 (8.6)	**<0.001**
Controls	204,509	15,803 (7.7)		33,173 (16.2)		22,335 (10.9)		4028 (2.0)	

* Patients who had neither pertussis nor coughing illness. ** Values < 10 per cell. Statistically significant *p*-values are in bold font. COPD, chronic obstructive pulmonary disease; CVD, cardiovascular disease.

**Table 3:** Rate of pertussis complications (any), by age group.

Age Group	Patients with Pertussis, *n*	Patients with Pertussis Who Had Complication/s (Any), *n*	Rate per 1000 Cases	*p*-Value
18–44 y	614	19	30.9	Ref
45–64 y	626	34	54.3	***p*** **= 0.042**
65+ y	548	25	45.6	*p* = 0.191

Statistically significant *p*-value is in bold font.

**Table 4:** Prescription rate per 1000 patients, 2008–2019, all ages.

All Ages (2008–2019)		Prescription Rate per 1000 Among Cases	Prescription Rate per 1000 Among Controls	Rate Ratio (Pertussis Cases vs. Controls)
	Asthma and COPD medication	9269.1	6951.1	1.33 (1.29, 1.37)
	CVD medication *	32,300.8	22,646.4	1.43 (1.40, 1.46)
	Diabetes medication	13,189.1	7895.3	1.67 (1.61, 1.73)
	Obesity medication	1256.4	1157.3	1.09 (0.95, 1.25)

COPD, chronic obstructive pulmonary disease; CVD, cardiovascular disease. * CVD medication excluded in 18–44y age group due to values of <10 per cell.

**Table 5:** The estimated costs associated with pertussis in adults.

Item	Type of Patient, (N)	Mean Number per Case	Out-of-Pocket Costs—Each GP Visit (Standard GP Consultation) per Case	*Total Estimated Out-of-Pocket Costs (Due to Pertussis) per Case*	Government Costs—Each GP Visit (Standard GP Consultation) per Case	*Total Government Costs per Case*	*Grand Total Cost per CASE (Out-of-Pocket + Government Cost)*	*Total Estimated Cost Per Year* *(N Cases ^#^ x Cost Per Case )*
**GP visits (cases with no complication)**	Bulk billing patient, (102)	9 visits	AUD 0	AUD 0	AUD 41 *	AUD 369	**AUD 369**	AUD 37,638
	Non-bulk billing patient, (47)	9 visits	AUD 44 *	AUD 396	AUD 41 *	AUD 369	**AUD 765**	AUD 35,955
**Laboratory tests**	Bulk billing patient, (135)	1 test	AUD 0	AUD 0	AUD 41 **	AUD 41	**AUD 41**	AUD 5535
	Non-bulk billing patient, (14)	1 test	AUD 40 **	AUD 40	AUD 41 **	AUD 41	**AUD 81**	AUD 1134
		
**Antibiotic prescription (for treatment) ^# #^**	All patients (149)	2 scripts	AUD 31.60 ***	AUD 63.20	AUD 0	n/a	**AUD 63.20**	AUD 9417
		
**Grand total cost (cases with no complication)**	Bulk billing patient, (102)						**AUD 473.20**	AUD 48,267
	Non-bulk billing patient, (47)						**AUD 909.20**	AUD 42,733
	All patients							**AUD 91,000**

* Service | Medical Costs Finder | Australian Government Department of Health [29]. ** Service | Medical Costs Finder | Australian Government Department of Health [28]. *** Pharmaceutical Benefits Scheme (PBS)|Price Premiums [27]. ^#^ Estimated average number of cases per year = 149; annual estimates were calculated based on out-of-pocket service rates, using 31% for GP visits [29] and 9% for laboratory testing [28]. ^# #^ Data was not included for repeat medication.

## Data Availability

The datasets created during and/or analysed in the current study are not publicly available due to legal constraints.

## Supplementary Materials

The following supporting information can be downloaded at: https://www.mdpi.com/article/10.3390/vaccines13101029/s1, Table S1. Lists of coughing illnesses; Table S2. Lists of complications; Table S3. Number of patients with comorbidities and rate per 1000 among cases and controls, all ages; Table S4. Number of prescriptions issued among pertussis cases by age group and comorbidity, 2008–2019; Table S5. The estimated costs associated with pertussis in adults who had complications; and Figure S1. Laboratory tests requested by GPs among pertussis cases by year, 2009–2019.
