# Supplementary material for: The Burden of Pertussis Disease and Vaccination Coverage in Australian Adults Attending Primary Health Care
Source: Vaccines (Basel). 2025 Oct 2;13(10):1029. doi: 10.3390/vaccines13101029 (PMC12568010; doi:10.3390/vaccines13101029)
Supplement: Supplementary file 1 [file vaccines-13-01029-s001.zip › vaccines-3854792-supplementary tables.pdf]

Supplementary Tables

Table S1. Lists of coughing illnesses

Chronic cough
Bacterial bronchitis
Chronic bronchitis
Eosinophilic bronchitis
Recurrent bronchitis
Complicated bronchitis
Viral bronchitis
Laryngotracheobronchitis
Post bronchitis cough
Chronic Sino-bronchitis
Post infective cough
Post bronchitis cough
Croupy cough
Post viral cough
Wheezy bronchitis

Table S2. Lists of complications

Encephalitis
Seizures
Pneumonia
Rib fracture
Subdural haemorrhage
Syncope
Urinary incontinence
Rectal prolapse

Table S3. Number of patients with comorbidities and rate per 1,000 among cases and controls, all ages.

	Asthma or COPD		CVD		Diabetes		Obesity	
	n, (Rate /1,000 Patients)	OR 95%CI, p-value	n, (Rate /1,000 Patient)	OR 95%CI, p-value	n, (Rate /1,000 Patients)	OR 95%CI, p-value	n, (Rate /1,000 Patients)	OR 95%CI, p-value
Pertussis cases	446 (249.4)	-	246 (140.4)	-	201 (112.4)	-	156 (87.3)	-
Controls	31,856 (43.8)	-	43,859 (60.3)	-	39,718 (54.6)	-	21,170 (29.1)	-
Cases vs controls		OR=7.25 (6.51-8.08) **<0.001**		OR=2.54 (2.22-2.91) **<0.001**		OR=2.19 (1.89-2.54) **<0.001**		OR=3.19 (2.70-3.76) **<0.001**

Footnote:
OR, odds ratio; n, number of patients; COPD, chronic obstructive pulmonary disease; CVD, cardiovascular disease.
Statistically significant p-values are in bold font.

Table S4. Number of prescriptions issued among pertussis cases by age group and comorbidity, 2008–2019.

	Asthma and COPD medication	CVD medication	Diabetes medication	Obesity medication
**18–44 years**				
Total number of prescriptions issued (n)	624	*	128	84
Average number of prescriptions per person (n)**	5	*	8	2
**45–64 years**				
Total number of prescriptions issued (n)	1185	979	655	80
Average number of prescriptions per person (n)**	8	19	11	1
**65+ years**				
Total number of prescriptions issued (n)	2325	6967	1868	32
Average number of prescriptions per person (n)**	13	36	15	1

Footnote:
COPD, chronic obstructive pulmonary disease; CVD, cardiovascular
* Values < 10 per cell. ** Rounded to the nearest whole number.

Table S5. The estimated costs associated with pertussis in adults who had complications

**Item**	**Type of patient**	**Mean number per case**	**Out-of-pocket costs - each GP visit (standard GP consultation) per case**	***Total estimated out-of-pocket costs (due to pertussis) per case***	**Government costs- each GP visit (standard GP consultation) per case**	***Total government costs per case***	***Grand total cost Per CASE (out-of-pocket + government cost)***
**GP visits (cases with complications)**	Bulk billing patient	12 visits	$0	$0	$41*	$492	$492
	Non-bulk billing patient	12 visits	$44*	$528	$41*	$492	$1,020
**Laboratory tests**	Bulk billing patient	1 test	$0	$0	$41**	$41	$41
	Non-bulk billing patient	1 test	$40**	$40	$41**	$41	$81
**Antibiotic Prescription (for treatment)** # #	Patients (all)	2 scripts	$31.60***	$63.20	$0	n/a	$63.20
**Grand total cost (cases with complications)**	Bulk billing patient						**$596.20**
	Non-bulk billing patient						**$1,164.20**

Source:
* Service | Medical Costs Finder | Australian Government Department of Health [29]. ** Service | Medical Costs Finder | Australian Government Department of Health [28]. *** Pharmaceutical Benefits Scheme (PBS) | Price Premiums [27].
# # Data was not included for repeat medication.
