# Supplementary figures and images for: The Burden of Pertussis Disease and Vaccination Coverage in Australian Adults Attending Primary Health Care
Source: Vaccines (Basel). 2025 Oct 2;13(10):1029. doi: 10.3390/vaccines13101029 (PMC12568010; doi:10.3390/vaccines13101029)

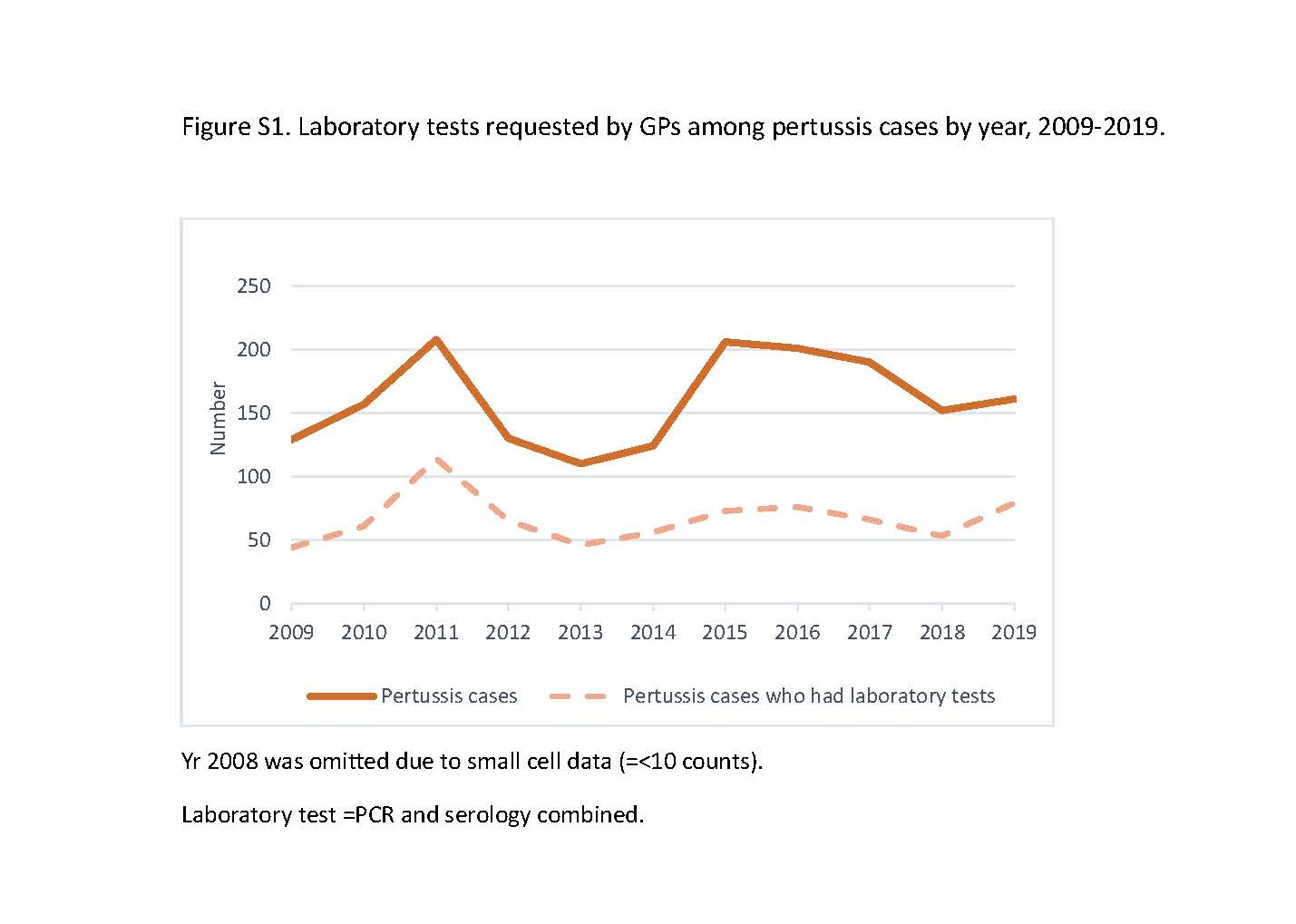

Supplement: Supplementary file 1 [file vaccines-13-01029-s001.zip › Supplementary figure.jpg]
